# Multi-omics characterization of β-myrcene-evolved *Pseudomonas* sp. M1 reveals convergent FleQ mutations and altered catabolic efficiency

**DOI:** 10.3389/fmolb.2026.1800048

**Published:** 2026-04-13

**Authors:** Filipa Soares, Rafaela Roque, Patrick Hellwig, Dirk Benndorf, Pedro M. Santos

**Affiliations:** 1 CBMA Molecular and Environmental Biology Centre, University of Minho, Braga, Portugal; 2 Otto von Guericke University, Bioprocess Engineering, Magdeburg, Germany; 3 Max Planck Institute for Dynamics of Complex Technical Systems, Bioprocess Engineering, Magdeburg, Germany; 4 Applied Biosciences and Process Engineering, Anhalt University of Applied Sciences, Microbiology, Köthen, Germany

**Keywords:** adaptive laboratory evolution, FleQ, metabolism, monoterpenes, proteomics, *Pseudomonas* sp. M1

## Abstract

β-Myrcene is a high-value monoterpene precursor whose high hydrophobicity limits microbial biotransformation. In aqueous medium, β-myrcene forms droplets that *Pseudomonas* sp. M1 accesses through chemotaxis mediated by the genomic island (GI)-encoded methyl-accepting chemotaxis protein MyrS. To identify genetic targets for strain improvement, we subjected M1 to adaptive laboratory evolution (ALE) for 600 generations under β-myrcene selection and characterized two evolved isolates, M2C19 and M3C22, using comparative genomics, quantitative proteomics, and metabolite profiling. Both lineages independently acquired mutations in the AAA + ATPase domain of FleQ, the master regulator of flagellar biosynthesis, resulting in loss of polar flagella and Tad pilus proteins, and strong reduction of chemotaxis signal transduction (CheA, CheW), putatively impacting response to β-myrcene chemoattractant signal. Despite identical growth rates during exponential phase, evolved strains achieved ∼33% higher final OD_600_ than wild-type M1. Metabolite analysis indicated enhanced pathway flux: M2C19 accumulated myrcenoic acid 10.6-fold above wild-type, while M3C22 accumulated 3.5-fold, and upstream intermediates (myrcen-8-ol, myrcenal) were depleted in both strains. Proteome profiling revealed distinct temporal dynamics of GI induction: M2C19 showed early upregulation of GI proteins, whereas M3C22 displayed delayed induction at early exponential phase with recovery by late exponential phase. Beyond the GI, both evolved strains converged on reduced motility/chemotaxis systems and extensive membrane remodeling, while core metabolic processes diverged. M2C19 broadly upregulating respiration and β-oxidation components, and M3C22 showing systematic downregulation of these pathways at early growth stages. Overall, the results identify FleQ as a major adaptive target during β-myrcene-driven evolution and reveal distinct proteometabolic strategies that improve monoterpene processing under laboratory selection.

## 1 Introduction

β-Myrcene is an acyclic monoterpene present in a wide range of plant essential oils and commonly used as a precursor for high-value terpenoids, fragrances, and elastomers (Hornung et al., 2017). However, the physicochemical properties of β-myrcene including its low aqueous solubility and strong hydrophobicity, pose major challenges for microbial uptake, often leading to membrane perturbation and oxidative stress that limit bioavailability and conversion efficiency (López-Maury et al., 2008; Santos and Sá-Correia, 2009; Lee and Palsson, 2010).

Among the microorganisms capable of degrading monoterpenes, *Pseudomonas* sp. M1 has emerged as an exceptional model for studying β-myrcene metabolism and adaptation. This strain was originally isolated from sediments due to its ability to use β-myrcene as sole carbon and energy source (Iurescia et al., 1999). M1 metabolizes β-myrcene through an oxidative pathway encoded within a 28-kb β-myrcene genomic island (GI), which allows β-myrcene uptake and catabolism through a coordinated network of enzymes, transporters, and regulators (Iurescia et al., 1999; Santos and Sá-Correia, 2007; Santos and Sá-Correia, 2009; Soares-Castro and Santos, 2014; Soares-Castro et al., 2017). The GI is organized into eight transcriptional units (TUs 1–8) that direct successive oxidative and regulatory processes during growth on β-myrcene. TU5 encodes the enzyme that catalyzes the initial oxidation step, converting β-myrcene into myrcen-8-ol, a crucial step for the metabolism of this compound since it triggers the initial activation of the GI. Together with TU6 and TU3, β-myrcene is converted into myrcenal, and myrcenoic acid. These metabolites hold significant industrial interest due to their potential application in the production of aroma compounds and biodegradable materials (Soares-Castro and Santos, 2014; Soares-Castro et al., 2017). TU1 and TU7 also comprise catabolic genes, encoding proteins such as CoA dehydrogenases and CoA hydratases, being related with the last steps of β-myrcene catabolism before entering the central metabolism. A summarized overview of this pathway is provided in Supplementary Figure S1 (Supplementary Material). TU4 and TU8 encode regulatory elements, including LuxR-type transcriptional regulators, being responsible for the regulation of GI expression. TU2 encodes a methyl-accepting chemotaxis protein (MyrS) that enables M1 to sense β-myrcene and possibly other terpenes (Soares-Castro and Santos, 2014). β-myrcene partitions into discrete droplets in liquid culture. *Pseudomonas* sp. M1 chemotactic capacity allows cells to actively locate and aggregate at these droplets, effectively overcoming the bioavailability limitation imposed by substrate hydrophobicity.

*Pseudomonas* sp. M1 has a remarkable metabolic flexibility. Metabolomic and transcriptomic analyses have hinted on the sequential activation of the referred transcriptional units and the accumulation of the oxidation intermediates mentioned above (Soares-Castro and Santos, 2014; Soares-Castro et al., 2017). The key enzyme myrcene hydroxylase (MyrH) encoded in TU5, catalyses the initial oxidation of β-myrcene, while several oxidoreductases and aldehyde dehydrogenases complete its transformation into myrcenoic acid. These intermediates hold strong biotechnological potential as renewable precursors for fine chemicals and biodegradable materials, demonstrating the potential of *Pseudomonas* sp. M1 as a versatile biocatalyst for sustainable industrial applications. However, β-myrcene exerts substantial hydrophobic and oxidative stress, challenging cellular homeostasis and membrane integrity (Santos and Sá-Correia, 2009; Esmaeili and Hashemi, 2011; Soares-Castro et al., 2023). Therefore, efficient biotransformation of β-myrcene also requires the coordinated activation of catabolic enzymes, stress-response proteins, and envelope-modification pathways (Santos and Sá-Correia, 2009; Soares-Castro and Santos, 2014).

In this context, adaptive laboratory evolution (ALE) was performed under β-myrcene selective pressure, seeking for genetic trait targets leading to enhanced biotransformation performance. After prolonged cultivation, evolved isolates with improved growth and β-myrcene conversion were isolated and their genome sequence determined. To further characterize them, we combined comparative genomics, label-free quantitative LC–MS/MS proteomics and GC–MS analysis of β-myrcene biotransformation products, aiming to characterize the β-myrcene-dependent proteome dynamics of wild-type versus evolved lineages, and associated metabolite production. Altogether, the results described here illustrate how evolved M1 lineages coupled catabolic specialization with regulatory reorganization and structural remodeling to enhance their β-myrcene biotransforming capacity.

## 2 Materials and Methods

### 2.1 Strains, media, and growth conditions

*Pseudomonas* sp. M1 wild-type (hereafter referred to as M1), and evolved isolates were routinely maintained on LB agar and grown in mineral medium (MM - 8.9 mM K_2_HPO_4_, 6.2 mM NaH_2_PO_4_, 34.2 μM EDTA, 7 μM ZnSO_4_, 6.8 μM CaCl2, 18 μM FeSO_4_, 0.8 μM Na_2_MoO_4_, 0.7 μM CuSO_4_, 1.7 μM CoCl_2_, 1.9 μM MnCl_2_, 15.1 mM (NH_4_)2SO_4_ and 0.5 mM MgCl_2_) supplemented with either lactate (48 mM) or β-myrcene (CAS number: 123–35-3; density: 0.794 g/mL; water solubility 5.6 mg/L at 25 °C, partition coefficient octanol/water (log P_ow_) 4.82 at 30 °C) as the sole carbon and energy source. β-myrcene was added directly to cultures at 100 µL per flask that assures a constant water-phase saturation.

### 2.2 Adaptive laboratory evolution

For the ALE experiments, all cultures were initiated from a single *Pseudomonas* sp. M1 colony. A total of five independent cultures (200 mL) were propagated in mineral medium supplemented with β-myrcene as the sole carbon source. Cultures were grown in 500-mL Erlenmeyer flasks with rubber stoppers to maintain vapor–liquid equilibrium and limit β-myrcene volatilization, at 30 °C and 180 rpm (orbital shaking). Cultures were transferred every 24 h using 20-fold dilutions. The final OD600 nm (OD) and pH were measured every day, before the propagation step. Colony forming units (CFUs) were counted every week to control the number of generations reached by each culture and control for possible contaminations. Moreover, triplicates of each culture were stored at −80 °C, every 50 generations. After 600 generations, 50 evolved isolates from each culture were obtained by plating into *Pseudomonas* Isolation Agar (PIA). 80% of the isolates evidenced enhanced antibiotic resistance (kanamycin (>50 μg/mL) or gentamicin (>30 μg/mL)) and were discarded for further analysis in the context of the present study. The plasmid pSEVA637-P5 (promoter probe using Green Fluorescent Protein (GFP) as reporter and with promoter P5 that controls the expression of the first catabolic enzyme (myrcene hydroxylase of the GI) (Soares-Castro et al., 2017) was transferred to selected evolved isolates. Promoter activity was quantified by measuring GFP expressed from pSEVA637-P5, with fluorescence determined using a Qubit fluorometer (Thermo Fisher Scientific), and expressed as fold change (evolved/M1) to identify evolved isolates with GI-related enhanced promoter activity, as a hint of higher β-myrcene catabolism. Each daily transfer used 10 mL of culture into 190 mL fresh medium (20-fold dilution), and 100 µL β-myrcene was added at each refresh to maintain saturation.

### 2.3 Whole-genome sequencing

The genomic DNA of evolved strains and M1 was extracted, processed according to Illumina instructions to generate Nextera XT paired-end libraries (2x150 bp) and sequenced using high-throughput Illumina Hiseq platform, as a paid service. The obtained datasets were firstly trimmed with Trimmomatic version 0.36. The program scanned all the reads with a 2-base wide sliding window, cutting when the average quality per base dropped below 20 (Leggett et al., 2013). For the scanning of genetic variants between M1 strain (NZ_CP094343.1) and the evolved cultures and isolates, Breseq (Deatherage and Barrick, 2014) version 0.39.0 was used. For the evolved cultures, polymorphism-prediction was used since the samples were not expected to be clonal. For the isolated evolved strains clonal mode was used. Results of Breseq-based comparative analysis were cross-validated with bcftools.

### 2.4 Protein extraction

Whole-cell protein extracts were obtained from M1 and evolved isolates (M2C19 and M3C22). Strains were grown overnight in MM supplemented with either lactate or β-myrcene at 30 °C with agitation (180 rpm). Cultures were refreshed and grown to OD = 0.5 (early exponential phase) or OD = 0.8 (late exponential phase). Cells were pelleted (10,000 × g, 10 min, 4 °C), resuspended in Urea Lysis Buffer (8 M urea, 50 mM Tris-HCl pH 6.8, 10% glycerol, 2% DTT, 10% SDS), and lysed by 15 × 10 s sonication cycles. Lysates were clarified by centrifugation and stored at −80 °C until quantification. All conditions were analyzed in three independent biological replicates.

### 2.5 Protein quantification and SDS–PAGE

Protein concentration was determined using the modified Lowry method with BSA as a standard. After precipitation with deoxycholate (DOC)/trichloroacetic acid (TCA), pellets were resuspended, reacted with Folin–Ciocalteu reagent, and measured at 750 nm. For quality assessment, 300 µg of total protein per sample were resolved by 10% SDS–PAGE following Laemmli’s protocol (He, 2011) and visualized with Coomassie Brilliant Blue G-250.

### 2.6 LC–MS/MS proteomics workflow

#### 2.6.1 Experimental design

Proteomic analysis was performed on 36 samples comprising three strains (wild-type M1, evolved isolates M2C19 and M3C22), two carbon sources (48 mM sodium lactate or β-myrcene as sole carbon source), and two growth phases (OD 0.5, exponential phase; OD 0.8, late exponential phase), with three biological replicates per condition (3 strains × 2 carbon sources × 2 growth phases × 3 replicates = 36 samples).

#### 2.6.2 Sample preparation and NanoLC–MS/MS acquisition

The protein was tryptic digested using FASP digestion with 25 µg protein (Heyer et al., 2019). The samples were dried in speed vacuum overnight and resuspended in 75 μL of Load A Buffer (0.1% Trifluoracetic acid (TFA) LC/MS grade). The peptides were analysed by LC-MS/MS using an UltiMate® 3,000 nano splitless reversed-phase nanoHPLC (Thermo Fisher Scientific, Dreieich) coupled online to a timsTOF™ pro 1 mass spectrometer (Bruker Daltonik GmbH, Bremen). The peptides (1 µg per sample) were trapped on a trap column (Dionex Acclaim, nano trap column, 100 μmi.d. x 2 cm, PepMap100 C18, 5 μm, 100 Å, nanoViper) and separated on a Dionex Acclaim PepMap C18 RSLC Nano-reversed phase column (2 μm particle size, 100 Å pore size, 75 μm inner diameter, and 500 mm length). The Mobile phase A was 100% water containing 0.1% formic acid (FA), and mobile phase B was 100% acetonitrile containing 0.1% FA. The gradient length for the separation was 120 min (Heyer et al., 2019). The timsTOF was operated in positive DDA-PASEF mode (Meier et al., 2018).

#### 2.6.3 Mass spectrometry data processing

Raw mass spectrometry data were processed using the FragPipe computational pipeline (v23.1) with the following components: MSFragger (v4.4) (Kong et al., 2017) for database searching against the *Pseudomonas* sp. M1 proteome (NZ_CP094343.1), 6,145 protein sequences plus common contaminants and reversed decoys), Philosopher (v5.1.2) for statistical validation using PeptideProphet and ProteinProphet, and IonQuant (v1.11.18) (Yu et al., 2021) for label-free quantification using the MaxLFQ algorithm. The parameters used in Fragpipe workflow are provided as Supplementary Material (MS_parameters).

#### 2.6.4 Protein quantification and normalization

Protein abundances were quantified using MaxLFQ intensity values (Yu et al., 2021). Zero values were treated as missing data. Raw intensities were log2-transformed and normalized by median centering across samples to correct for systematic differences in total protein loading. In total, 3,521 proteins were detected, normalized, and used for downstream analysis.

#### 2.6.5 Differential expression analysis

Differential protein expression was analyzed using DEqMS (v1.28.0), which implements an empirical Bayes method that weights variance estimation by the number of quantified peptides per protein, providing improved statistical power for proteomics data with heterogeneous peptide coverage. The analysis was performed in R (v4.5.2) using limma (v3.66.0) as the underlying framework. For each pairwise contrast, proteins were required to have non-missing values (complete cases) in each comparison group. In total, 18 contrasts (9 contrasts per carbon source) were analyzed resulting in a panel of 957 complete cases. Proteins detected exclusively in one group (presence/absence proteins) were reported separately, for qualitative analysis.

#### 2.6.6 Significance thresholds and classification

Proteins were classified as differentially expressed using the following criteria:| log_2_FC | ≥ 1.0 (corresponding to ≥2-fold change (FC)) and Benjamini–Hochberg adjusted p-value <0.05. Qualitative changes (presence/absence): Proteins detected in 3 of 3 replicates in one strain but completely absent (0 detections) in the comparison strain were classified as “gained/present” (present only in evolved) or “lost/absent” (present only in M1).

#### 2.6.7 Functional annotation

Curation of protein functional annotations was performed using eggNOG-mapper v2.1.13 against the eggNOG v5.0 database. Proteins were assigned to COG (Clusters of Orthologous Groups) functional categories. Furthermore, for protein contextualization, Operon Mapper was used for genome wide prediction of operon prediction (Taboada et al., 2018). The curated annotation is provided in Supplementary Material (Supplementary tables). For visualization, proteins were grouped into six major functional categories: Motility/Chemotaxis (COG category N and flagellar/chemotaxis-related terms), Energy Metabolism (C), Stress/Detoxification (O), Membrane/Transport (M, P, U, V), Transcription/Regulation (K, T), and Other/Unknown. Membrane-associated proteins were further curated using Gene Ontology terms for membrane localization (GO:0016020, GO:0016021, GO:0005886, GO:0009279). Subcellular localization was validated using PSORTb v3.0.

#### 2.6.8 Data visualization

Heatmaps were generated using matplotlib (v3.6.3) in Python and pheatmap (v1.0.13) in R, with hierarchical clustering (Euclidean distance, complete linkage) where applicable. Stacked bar plots and composite figures were generated using matplotlib with custom Python scripts. Volcano plots were generated using ggplot2 (v4.0.1) with ggrepel (v0.9.6) for non-overlapping gene labels. Multi-panel figures were assembled using patchwork (v1.3.2) in R. Multidimensional scaling (MDS) was performed in R using cmdscale () on Euclidean distance matrices of log2-transformed protein abundances. PERMANOVA was performed using the adonis2 () function from the vegan package (v2.7.2). Data manipulation in R used tidyverse packages (dplyr, tidyr). Color schemes used RColorBrewer palettes where applicable.

### 2.7 Gas chromatography–mass spectrometry (GC–MS)

Metabolite extraction and analysis were conducted as described by Soares-Castro et al. (2017). Cultures (50 mL) grown with β-myrcene were extracted with ethyl acetate, dried under nitrogen, and resuspended in hexane. GC–MS analyses were performed on an Agilent 7890A GC–5975C MSD using an HP-INNOWax column (30 m × 0.25 mm, 0.25 µm). The oven was programmed from 60 °C (1 min) to 250 °C at 10 °C min^-1^. Compounds were identified by mass spectra, retention indices, and comparison with commercial standards. Relative metabolite abundance was determined from integrated peak areas for comparative evaluation.

### 2.8 Statistical analysis

For non-proteomics assays (promoter activity, growth curves, metabolite fold-changes, and motility), values are reported as mean ± SD of biological replicates as indicated in the corresponding figure legends (typically n = 3).

## 3 Results

### 3.1 ALE of *Pseudomonas* sp. M1 reveals convergent regulatory mutations and enhanced β-myrcene utilization

**FIGURE 1:**
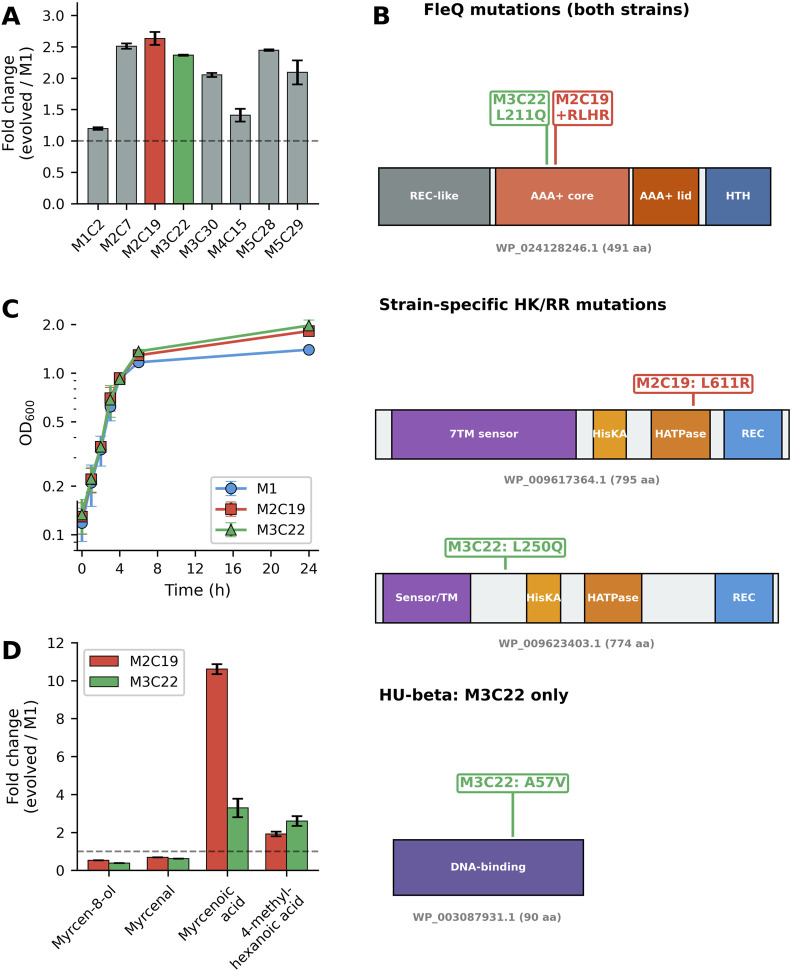
Characterization of evolved *Pseudomonas* sp. M1 strains **(A)** P5 promoter activity (fold change relative to M1) across evolved isolates from independent ALE lineages. M2C19 and M3C22 (highlighted) were selected for detailed characterization. Dashed line indicates M1 reference level (fold change = 1). 3 biological replicates of isolate cultures upon growth in β-myrcene during 24 h were used. Error bars represent standard deviation (SD) from biological replicates **(B)** Domain architecture of proteins harboring mutations in M2C19 and M3C22 evolved strains. FleQ (WP_024128246.1) contains mutations in both strains: L211Q in M3C22 and +RLHR insertion at position 222 in M2C19, both located in the AAA + ATPase core domain. Strain-specific mutations were identified in two histidine kinase/response regulator proteins: WP_009617364.1 (7TM sensor domain protein with L611R in M2C19) and WP_009623403.1 (sensor/TM domain protein with L250Q in M3C22). HU-beta (WP_003087931.1) carries an A57V mutation exclusively in M3C22. **(C)** Growth kinetics on β-myrcene as sole carbon source (log scale). Exponential growth rates were indistinguishable between strains, but evolved strains reached ∼33% higher final biomass at 24 h **(D)** Metabolomic profiling of β-myrcene pathway intermediates. Bar chart represents the fold-change of key catabolic intermediates (myrcen-8-ol, myrcenal, 4-methyl-hexanoic acid and myrcenoic acid) in evolved strains relative to wild-type M1, determined by GC-MS analysis at late exponential phase (OD 0.8). Error bars represent standard deviation (SD) from biological replicates.

To enhance β-myrcene biotransformation capacity of *Pseudomonas* sp. M1, 5 independent cultures (M1, M2, M3, M4, M5) of this strain were subjected to adaptive laboratory evolution (ALE) under continuous β-myrcene selective pressure, during 600 generations. 50 isolates from each evolved culture were then retrieved. Among the evolved isolates, eight were selected for further analysis. First, the promoter activity controlling the expression of the key enzyme of the GI, myrcene hydroxylase, was assessed (as described in Materials and Methods section). The results of this analysis are depicted in Figure 1A. While evolved isolates M1C2 and M4C15 evidenced only a slight increase (<1.5 fold) of P5 promoter activity when compared to the wild-type strain M1, the other evolved isolates presented an increased P5 promoter activity, between 2 and 2.5-fold, upon 24 h of cultivation in β-myrcene, suggesting that the accumulated mutations led to enhancement of β-myrcene catabolic performance. Therefore, the genome of all eight evolved strains was sequenced and the accumulated mutations inferred using Breseq. The complete results of this analysis, corroborated by bcftools analysis, are in Supplementary Table S1. Strikingly, comparative genomics revealed that seven out of the eight selected strains (only exception was M3C30) acquired mutations in the *fleQ* gene (PM1_RS22200, WP_024128246.1), encoding a σ^54^-dependent master regulator of flagellar motility, biofilm formation, and envelope biogenesis (Jyot et al., 2002; Jain and Kazmierczak, 2014; Matsuyama et al., 2016; Molina-Henares et al., 2017). Moreover, M2C7, M5C28 and M5C29 evolutionary paths resulted in hypermutator strains (as a consequence of mutations in *mutL*). In agreement, all these 3 strains accumulated between 73–102 mutations whereas the other isolates accumulated between 2 and 8 mutations. Based on this comparative genomics analysis coupled with P5 promoter activity, we decided to focus our attention on M2C19 and M3C22 evolved strains taking into consideration: i) their lower number of accumulated mutations; ii) presence of *fleQ* mutation; iii) max enhancement of P5 promoter activity, among non-hypermutator strains (since hypermutators do hold enhanced genetic instability harder to control).

In M3C22, *fleQ* harbors a single nucleotide polymorphism resulting in an L211Q substitution within the AAA + ATPase core domain (Walker A motif), while in M2C19 a 12-bp insertion encoding four additional amino acids (+RLHR) at position 222 (Figure 1B). Both mutations are localized on the functionally critical AAA + ATPase core, a region required for ATP-dependent oligomerization, conformational remodeling, and σ^54^-dependent transcriptional activation, indicating that these substitutions may directly impact the FleQ regulatory function (Shingler, 2011; Matsuyama, et al., 2016).

In addition to convergent *fleQ* mutations, strain-specific variants were identified, including genes encoding histidine kinase/response regulator (HK/RR) two-component systems. M2C19 acquired an L611R substitution in a 7TM (seven-transmembrane) sensor domain protein (PM1_RS26305, WP_009617364.1), whereas M3C22 carried an L250Q mutation in a sensor/TM (sensor/transmembrane) domain protein (PM1_RS20160, WP_009623403.1), as well as an A57V substitution in the global DNA-binding protein HU-beta (HupB, WP_003087931.1) (Figure 1B). Interestingly, all the detected mutations targeted intricate regulatory systems (FleQ and HupB) and signal transduction, suggesting the occurrence of parallel evolutionary trajectories of M2C19 and M3C22, converging on the modulation of critical cellular functions: transcriptional regulation and environmental sensing as key components of the adaptive response to β-myrcene biotransformation (Wadach, et al., 2025).

Growth curve analysis (Figure 1C) revealed identical exponential phase kinetics across strains (0–3 h): M1, μ = 0.54 h^-1^ (*R* ^2^ = 0.998); M2C19, μ = 0.55 h^-1^ (*R* ^2^ = 0.993); M3C22, μ = 0.54 h^-1^ (*R* ^2^ = 0.993). Despite equivalent growth rates during exponential phase, evolved strains reached a significantly higher OD at 24 h (OD 1.82 ± 0.07 for M2C19; 1.97 ± 0.16 for M3C22) compared to M1 (1.40 ± 0.05), corresponding to ∼33% higher OD at 24 h.

To evaluate the metabolic efficiency of the evolved lineages, gas chromatography-mass spectrometry (GC-MS) analysis was performed to quantify the following key intermediates of the β-myrcene catabolic pathway during the late exponential phase (OD 0.8): myrcenal, myrcen-8-ol, 4-methyl-hexanoic acid, and myrcenoic acid (the terminal carboxylated product prior to CoA ligation and β-oxidation). M2C19 accumulated myrcenoic acid at 10.6-fold higher levels than M1, while M3C22 accumulated only 3.5-fold higher levels (Figure 1D; Supplementary Table S2). The upstream intermediates myrcen-8-ol and myrcenal were effectively depleted in evolved strains, particularly in M2C19 (16% and 24% of M1 levels, respectively). This depletion is consistent with a faster turnover of upstream intermediates when compared to M1. Conversely, the rapid upstream conversion led to a substantial accumulation of myrcenoic acid, the terminal carboxylated intermediate, suggesting that particularly M2C19 excels in the first steps of β-myrcene biotransformation compared to M3C22 and M1. Therefore, despite M2C19 and M3C22 harbor a convergent regulatory mutation in *fleQ* gene, the registered differences in the metabolic rates suggest that the other accumulated mutations (specific for each strain) led to parallel evolutionary trajectories, both resulting in enhanced β-myrcene utilization.

To further investigate the observed differences in β-myrcene metabolism, proteomic analyses were performed. Multidimensional scaling (MDS) was used to assess differences in overall proteome composition between the wild-type strain (M1) and the evolved strains (M2C19 and M3C22) across different carbon sources and growth phases (Supplementary Figure S2, Supplementary Material). In general, for each carbon source and strain, proteome variation associated with growth phase did not show significant differences, indicating that the proteome remained largely stable. Moreover, samples segregated into two distinct clusters (Dim2) corresponding to β-myrcene (cluster below the 0 axis) and lactate (cluster above the 0 axis) cultures, with no overlap between C-source conditions, reflecting extensive proteomic reprogramming in response to substrate availability. This pronounced separation indicates an extensive reprogramming required to β-myrcene mineralization. Importantly, even under lactate growth conditions, the wild-type and evolved lineages segregated into distinct groups, indicating that adaptive mutations reshaped basal cellular physiology independently of β-myrcene exposure. PERMANOVA analysis confirmed that strain accounts for 10.8% of proteome variance (p = 0.002), with growth condition (7.8%, p = 0.001) and growth phase (5.5%, p = 0.004) also contributing significantly (Supplementary Table S3).

### 3.2 Divergent GI and PRP proteome dynamics in evolved strains during β-myrcene metabolism

**FIGURE 2:**
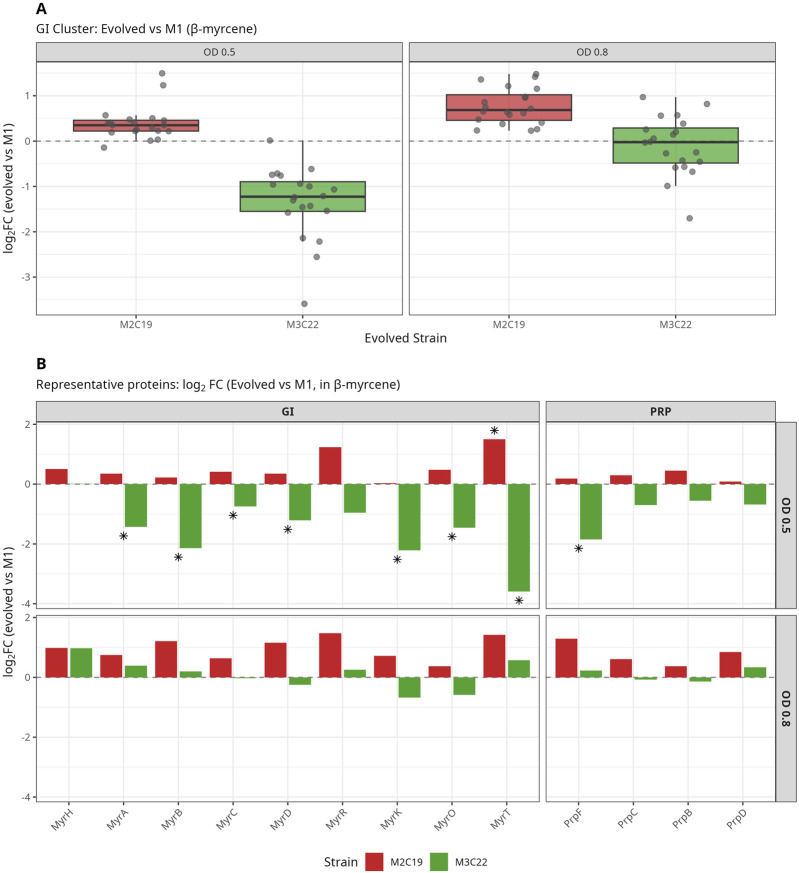
Differential expression of GI and PRP proteins in evolved strains relative to M1 during β-myrcene growth. **(A)** Distribution of log2 fold changes for all 21 GI cluster proteins in evolved strains (M2C19, M3C22) relative to wild-type (M1) during growth on β-myrcene. Boxplots show the median and interquartile range; individual proteins are overlaid as points. M2C19 (red) shows similar GI expression to M1 at both growth phases (median log_2_FC near 0). M3C22 (green) displays significantly reduced GI expression at OD 0.5 (median log_2_FC ∼ −1.2) but recovers to near-M1 levels by OD 0.8, indicating delayed induction of the β-myrcene catabolic pathway. **(B)** Log2 fold changes of representative GI and PRP cluster proteins. GI cluster proteins: MyrT (OmpW/AlkL family), MyrR (LuxR-family regulator), MyrH (myrcene hydroxylase), MyrA (aldehyde dehydrogenase), MyrB (alcohol dehydrogenase), MyrC (enoyl-CoA hydratase), MyrD (acyl-CoA dehydrogenase), MyrK (acetyl-CoA C-acyltransferase), MyrO (3-hydroxyacyl-CoA dehydrogenase); PRP operon: PrpF (2-methylisocitrate dehydratase), PrpC (2-methylcitrate synthase), PrpB (methylisocitrate lyase), PrpD (2-methylcitrate dehydratase). Asterisks (*) denote statistical significance (FDR <0.05, DEqMS).

The β-myrcene catabolic pathway (depicted in Supplementary Figure S1) requires the expression of proteins encoded by the β-myrcene-associated genomic island (GI), and propionate (PRP). Therefore, their expression profiles in evolved strains (M2C19 and M3C22) were compared against the wild-type M1 strain, during growth on β-myrcene (Figure 2). At early exponential phase, M2C19 expression profile was already slightly above M1 expression levels, particularly with higher levels for MyrT, putatively associated with β-myrcene internalization, MyrR, the master regulatory system in the GI, and MyrH, the key enzyme for initial β-myrcene oxidation. This enhanced abundance of GI-related proteins was even more pronounced at later exponential phase, including of the enzymes involved in the lower pathway branches (e.g., MyrK and Prp-related (Figure 2)). In contrast, in early exponential phase, M3C22 showed a significantly lower expression of the GI-related proteins when compared to both M2C19 and M1, characterized by a median log_2_FC of ∼ −1.2 (Figure 2A). This aligns with registered ∼ -1 log_2_FC abundance of the master GI regulator MyrR. However, at late exponential, GI protein levels in M3C22 recovered to expression thresholds above or near-M1 levels (overall log_2_FC close to 0). Therefore, M3C22 exhibited a distinct temporal profile for GI protein abundance. At OD 0.5, multiple GI-encoded proteins showed reduced levels relative to both M1 and M2C19, including the methyl-accepting chemotaxis protein MyrS (log_2_FC −1.58 vs. M1). By OD 0.8, these differences were reversed, with M3C22 reaching wild-type or elevated expression levels (MyrS log_2_FC +0.56 vs. M1). In contrast, M2C19 showed elevated GI expression at both time points.

A detailed examination of the expression levels of representative proteins from the GI cluster and the PRP operon confirmed the different temporal patterns of GI induction for each evolved isolate (Figure 2B). At OD 0.5, M3C22 showed a statistically significant downregulation of nearly all key GI catabolic enzymes. Notably, the PRP operon (essential for processing propionyl-CoA generated during β-oxidation through the 2-methylcitrate cycle) followed an identical delayed induction pattern in M3C22, with PrpF showing significant initial downregulation (Figure 2B). Thus, while M2C19 evidences an early and enhanced induction of β-myrcene catabolic pathway, M3C22 induction of β-myrcene catabolic pathway seems to occur only upon an extended lag-like period. The initial delayed expression of the GI and PRP clusters in M3C22 may reflect an initial constrain to assess β-myrcene and/or a lifestyle shift that precedes pathway stabilization, ultimately supporting enhanced β-myrcene biotransformation (Figure 1D).

Furthermore, the inspection of expression levels of GI and PRP clusters during cultivation in lactate as sole carbon source revealed that while in M1 there is a basal level of expression for most of the associated proteins, in both M2C19 and M3C22 such basal level seems to be either reduced or completely abolished, suggesting a tighter control of protein basal expression leakage in the evolved strains. This observation was also essential to take into account, as log_2_FC estimations of β-myrcene/lactate contrasts may be misleading due to different basal thresholds. Therefore, we opt to only analyze log_2_FC contrasts within same carbon source (comparing evolved vs. M1 strain) (Supplementary Figure S3 and, Supplementary Table S4).

### 3.3 Proteome-wide remodeling in evolved strains reveals convergent and divergent adaptive responses

**FIGURE 3:**
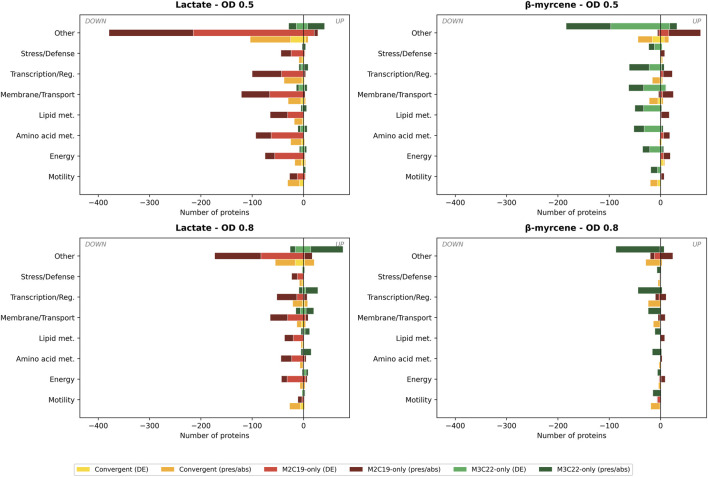
Functional categorization of proteome changes in evolved strains compared to wild-type M1. Diverging bar charts showing the distribution of differentially expressed proteins across seven COG-based functional categories under four growth conditions: Lactate OD 0.5, β-myrcene OD 0.5, Lactate OD 0.8, and β-myrcene OD 0.8 (D). Bars extending leftward indicate downregulated proteins; bars extending rightward indicate upregulated proteins. For each functional category, three stacked bar groups represent: convergent changes (proteins significantly altered in both M2C19 and M3C22 in the same direction; yellow for differential expression, orange for presence/absence), M2C19-specific changes (red for DE, dark red for presence/absence), and M3C22-specific changes (green for DE, dark green for presence/absence). Presence/absence patterns identify proteins uniquely detected (present) or completely absent in evolved strains relative to M1 (3/3 replicates required for detection, 0/3 for absence). Motility (COG N; flagellar assembly, chemotaxis); Energy (COG C; respiration, electron transport); Amino acid met. (COG E; biosynthesis and catabolism); Lipid met. (COG I; fatty acid metabolism); Membrane/Transport (COG M, P, U; transporters, porins, secretion); Transcription/Reg. (COG K, T; transcription factors, signal transduction); Stress/Defense (COG O, V; chaperones, oxidative stress response); Other (remaining COG categories and uncharacterized proteins). Details of this analysis are provided in Supplementary Table S5.

To capture the full scale of proteome remodeling in the evolved strains, the analysis of the proteomic data was expanded beyond the GI and PRP clusters. After excluding proteins encoded within the GI, PRP, and Liu clusters (previously analyzed in Figure 2; Supplementary Figure S3), 3,490 proteins (Supplementary Table S5) were assessed for significant changes (|log_2_FC| > 1.0, FDR <0.05) and their presence/absence patterns. Of these, 1,628 (46.6%) exhibited significantly altered expression (|log2FC| > 1, FDR <0.05) or presence/absence changes in at least one condition. The lactate OD 0.5 condition showed the most extensive remodeling (1,166 proteins), dominated by M2C19-specific downregulation (804 proteins, with 400 representing complete protein loss). Under β-myrcene, M3C22 displayed the predominant downregulation pattern (353 proteins, at OD 0.5), while M2C19 showed a distinct gain-of-function signature (75 proteins uniquely present). Convergent changes between both evolved strains were predominantly downregulation (152 of 165 convergent proteins at lactate OD 0.5), consistent with shared reductive adaptation. Motility-related proteins showed the highest proportion of altered expression (60.8%), followed by amino acid metabolism (51.3%) and energy metabolism (50.9%). The asymmetric x-axis scale in Figure 3 reflects the predominance of downregulation (70%–91%) over upregulation across all conditions.

Overall, convergent proteome alterations, particularly at early exponential growth and mostly directed towards downregulation or protein absence, were registered in both lactate and β-myrcene conditions. Interestingly, although ALE was conducted using β-myrcene as sole carbon source, protein level alterations in evolved strains were higher in lactate, suggesting that the applied selective pressure not only led to proteome alterations associated with coping with β-myrcene but actually reshaped strain behavior in different physiological contexts. Indeed, alterations in basal expression of the GI-related proteins, under lactate cultivation, was also detected (above). Strain-specific divergence was evident in the distribution of proteome alterations across carbon sources. M2C19 exhibited more pronounced changes during lactate growth, predominantly directed toward downregulation or complete expression shutdown. In contrast, M3C22 showed stronger proteomic shifts during β-myrcene growth, consistent with its delayed activation of catabolic pathways (Figure 2). At late exponential phase (OD 0.8), the number of differentially expressed proteins declined sharply in both strains, indicating partial convergence toward wild-type proteome states. This temporal pattern suggests that many proteome alterations in evolved strains represent fine-tuning of early-phase adaptive responses rather than permanent metabolic restructuring.

### 3.4 Phenotypic and proteomic basis of motility loss in evolved strains

**FIGURE 4:**
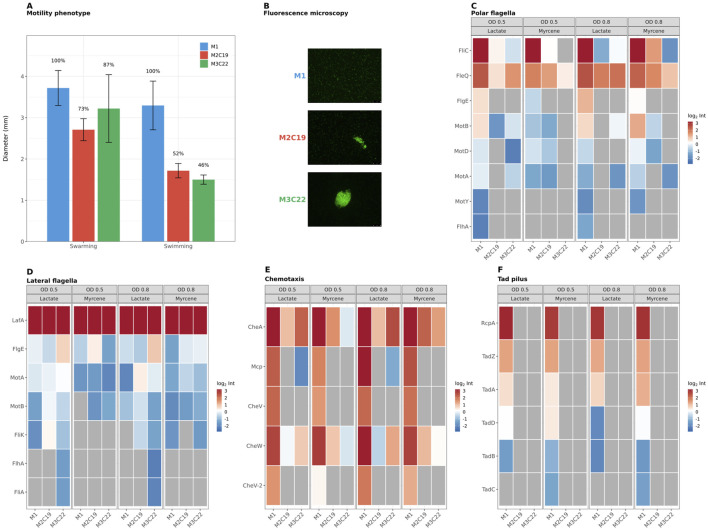
Motility phenotype and proteome alterations at level of motility and chemotaxis systems. **(A)** Motility phenotype assays. Bar plots showing swimming and swarming diameter (mm) for M1 (blue), M2C19 (red), and M3C22 (green). Error bars indicate standard deviation (n = 3). Percentages show motility relative to M1. **(B)** Fluorescence microscopy of M1 (wild-type), M2C19, and M3C22 strains grown on myrcene at OD 0.8. Cells carry plasmid pSEVA637-P5, a promoter probe expressing GFP under control of the P5 promoter that controls myrcene hydroxylase (MyrH) expression in the Genomic Island. Heatmaps **(C–F)** of normalized intensities for motility-related proteins across all experimental conditions. Only proteins with 3/3 valid replicates in a condition are displayed (grey = incomplete data). **(C)** Polar flagella proteins, including flagellin (FliC), motor proteins (MotA, MotB, MotD, MotY), basal body (FlgE), export apparatus (FlhA), and master regulator (FleQ). **(D)** Lateral flagella proteins, including lateral flagellin (LafA), motor proteins (MotA, MotB), and structural components (FlgE, FlhA, FliA, FliK). **(E)** Chemotaxis associated proteins, including histidine kinase CheA, coupling protein CheW, adaptor proteins CheV and CheV-2, and methyl-accepting chemotaxis protein Mcp. **(F)** Tad (tight adherence) pilus components, including RcpA, TadZ, TadA, TadB, TadC and TadD.

Taking into consideration the role of FleQ (mutated in both evolved strains) and the registered overall alterations of motility-related proteins (Figure 3), the swarming and swimming traits of evolved strains were evaluated. M2C19 and M3C22 exhibited a marked decrease in swimming (52% and 46% of M1, respectively) and swarming (73% and 87% of M1, respectively) diameters (Figure 4A). β-myrcene cultures of all three strains harboring pSEVA637-P5 (Soares-Castro et al., 2017) were also inspected, using fluorescence microscopy. Significantly, it was observed that M1 strain evidenced an almost even planktonic distribution (only very small aggregates were visualized). In contrast, M2C19 and M3C22 cultures were characterized by large cell aggregates, particularly in the case of M3C22, as illustrated in Figure 4B. This loss of motility and shift towards an aggregate lifestyle is aligned with the strongly reduced abundance of the polar flagellar machinery in the evolved proteomes (Figure 4C). While the master regulator FleQ showed only slight downregulation, its downstream targets were nearly all abolished. This includes the absence of the hook protein FlgE in both evolved strains. Consequently, essential structural components of the polar flagellum, such as the flagellin FliC and motor proteins (MotA, MotB, MotD, MotY), were either not detected or significantly downregulated across both C-sources (Figure 4C). These results reinforce the hypothesis that the identified *fleQ* mutations lead to a functional loss of its transcriptional activation properties, effectively decreasing polar flagella driven motility capacities.

Motility switch in evolved strains extends beyond the polar flagellar system, including the reconfiguration of other chemotaxis and adhesion-related systems. For instance, regarding the lateral flagellar system (Figure 4D), the proteomic response was mixed, with some components being retained or even upregulated (e.g., FhlA, FliA), although the main flagellin LafA remained at similar expression levels across the different conditions. The chemotaxis machinery (Figure 4E), particularly the core signal transduction proteins CheA and CheW, showed consistent lower abundance in evolved strains, supporting a stable reduction in the capacity to sense and respond to environmental stimuli and explaining the reduced directed motility.

Despite the loss of polar flagella and reduction of chemotaxis signal transduction components, the GI-encoded methyl-accepting chemotaxis protein MyrS (WP_409077363.1) remained expressed in both evolved strains, under β-myrcene conditions (Supplementary Table S4). In contrast, the core chemotaxis signal transduction components CheA and CheW were strongly downregulated, particularly under β-myrcene conditions (Figure 4E), suggesting a functional uncoupling, compromising β-myrcene sensing in the evolved strains.

The Tad (tight adherence) pilus complex (Figure 4F), which contributes to cell–cell interactions and surface attachment (Evans et al., 2025), was also largely absent in evolved strains, with most essential protein components remaining undetected.

The results outlined in Figure 4, suggest that M2C19 and M3C22 represent a compelling example of convergent evolution since both presented similar loss of motility associated functions, supported by consistent phenotypic and proteomic signatures. This is reinforced at the genetic level, as both lineages independently acquired mutations in *fleQ*, specifically targeting the same functional domain of this motility master regulator (Figure 1B). However, while the phenotypic outcome is convergent, a significant level of strain-specific divergent evolution is also evident (Figure 3), which likely dictates different adaptive, catabolic and metabolic responses observed during growth stages.

### 3.5 β-myrcene-associated cell envelope proteome dynamics

**FIGURE 5:**
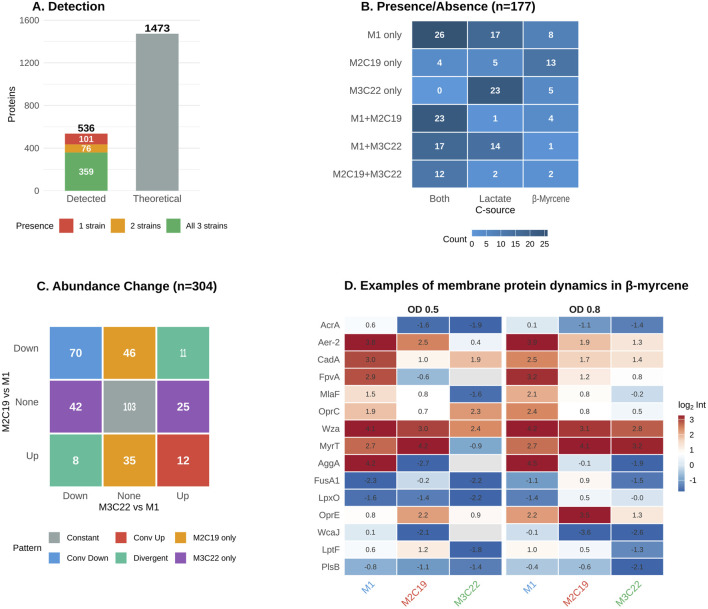
Membrane protein remodeling in evolved strains. **(A)** Detection overview showing the number of membrane proteins identified across the proteomics dataset. **(B)** Presence/absence heatmap (n = 177) showing strain-specific membrane protein detection patterns. Rows indicate strain combinations where proteins were detected; columns show carbon source specificity (Both = detected in both lactate and β-myrcene; Lactate = lactate-specific; Myrcene = β-myrcene-specific). **(C)** Abundance changes matrix (n = 304) for membrane proteins detected in all three strains (DEqMS data). Mosaic plot shows the relationship between M2C19 and M3C22 expression changes relative to M1 (|log_2_FC| ≥ 1 threshold). **(D)** Selected membrane protein examples under β-myrcene conditions (n = 15). Heatmap displays normalized log_2_ intensities for proteins representing key membrane functions: efflux (AcrA, FusA1), porins (OprE, OprC), the myrcene transporter MyrT (OmpW/AlkL family), lipid/LPS metabolism (LpxO, LptF, PlsB), and surface structures (Wza, CadA, MlaF).

The membrane proteome represents a critical interface between cellular physiology and adaptation to the highly hydrophobic environment imposed by β-myrcene. Therefore, the dynamics of this proteome fraction, particularly under β-myrcene growth conditions, was further investigated. First, we scanned the full genome of *Pseudomonas* sp. M1 using PSORTb. This approach allowed to select the membrane proteome fraction for which a significant classification as “Cytoplasmic Membrane” or “Outer Membrane”, resulting in a total of 1,473 membrane proteins (Figure 5A). The inspection of proteome MS data, resulted in the detection of 536 membrane proteins, representing approximately 36% of the theoretical membrane proteome. This detection rate reflects both the technical challenges inherent to membrane protein analysis and the dynamic nature of membrane protein expression under varying growth conditions. Notably, 359 proteins (67% of detected) were present across all three strains regardless of condition, establishing a conserved membrane protein core that likely serves essential housekeeping functions. The remaining 177 proteins exhibited strain-specific or condition-dependent detection patterns, representing the evolutionarily labile fraction of the membrane proteome where adaptive changes concentrate.

The distribution of strain-specific membrane proteins provides evidence for divergent evolutionary trajectories, mostly directed an apparent surface simplification (Figure 5B). Wild-type M1 retains 51 membrane proteins absent from both evolved strains, and the functional composition of these lost proteins is striking. Among them are multiple components of the c-di-GMP signaling network, including diguanylate cyclases and the LapD receptor, and chemotaxis receptors (PctC, multiple MCPs) and flagellar apparatus components (MotY, MotC, FlgH) (as also reported in Figure 4). The coordinated loss of these systems in both evolved lineages suggests strong selective pressure against energy-intensive surface structures and motility apparatus, aligning with growth as sessile aggregates on a lipophilic substrate that requires metabolic flux rewire. The pattern of proteins expressed exclusively in evolved strains includes both convergent trajectories and strain-specific expression signatures. Both M2C19 and M3C22 shared unique constitutive expression of 12 proteins, including the MexE multidrug efflux adaptor and an OmpA family protein (YfiB), consistent with enhanced efflux capacity as a shared adaptive response. Under β-myrcene-specific conditions, only the TolC family outer membrane protein (WP_446731348.1) and YjcH were found to be uniquely expressed in the evolved strains. However, the evolved strains also evidenced strain-specific expression signatures. M2C19 uniquely expresses 13 membrane proteins specifically under β-myrcene conditions, including an OprD family porin, the ExbB proton channel, and components of respiratory chain. M3C22, by contrast, shows 23 proteins detected exclusively under lactate conditions and only 5 under β-myrcene.

Among the membrane proteins quantifiable across all strains (Figure 5C), differential expression shows a striking 5.5-fold bias toward downregulation: 66 proteins (19%) exhibit convergent decreases in both evolved strains relative to M1, compared to only 12 (3%) with convergent increases. Therefore, both qualitative and quantitative patterns suggest a convergence of M2C19 and M3C22 on membrane protein remodeling (mostly towards simplification), despite the existence of strain-specific signatures.

The selected membrane proteins in Figure 5D provide some examples on how evolved strains have remodeled membrane composition and transport capacity, with putative implications for hydrophobicity and terpene permeability. The LPS export permease LptF and undecaprenyl-phosphate glucose phosphotransferase WcaJ both show convergent decreases (WcaJ: 4.6-fold reduction in M2C19). Reduced LPS content would alter the hydrophilic character of the outer membrane outer leaflet, potentially modifying terpene-membrane interactions. The convergent downregulation of the phospholipid trafficking protein MlaF, which maintains outer membrane lipid asymmetry, could lead to increased phospholipid content in the outer leaflet, creating a more hydrophobic surface barrier. Furthermore, rather than simply increasing efflux capacity, evolved strains have reconfigured their efflux systems. The canonical AcrA adaptor shows 5-fold convergent downregulation, but this seems to be compensated by selective expression of alternative components like FusA1 (4-fold upregulation in M2C19) and a TolC family protein (detected exclusively in evolved strains under myrcene). Together, these surface modifications suggest that both M2C19 and M3C22 have reshaped their cellular envelope for the specialized consumption of β-myrcene. These surface changes complement the previously observed motility loss (Figure 4), and shift from a motile/secretion-active phenotype to a sessile/aggregated phenotype (evolved strains), possibly providing an advantage for β-myrcene consumption.

### 3.6 Differential expression of respiratory and denitrification systems

**FIGURE 6:**
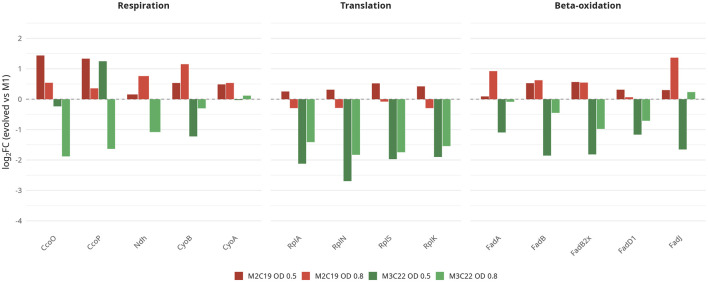
Divergent expression of core metabolic processes in evolved strains during β-myrcene catabolism. Bar plots of log_2_FC values between evolved strains and M1 for representative proteins from three functional categories: Respiration (CcoN, CcoP, Ndh, CyoB, CyoA); Translation (RplA, RplN, RplS, RplK); β-Oxidation (FadA, FadB, FadB2x, FadD1, FadJ). Data shown for two growth phases: OD 0.5 (early exponential) and OD 0.8 (late exponential). Missing log_2_FC values were not plotted. Full data is provided in Supplementary Table S5.

Notably, M2C19 and M3C22 constitutively expressed NADH-quinone oxidoreductase subunits NuoA and NuoL, while these subunits where not detected in M1, hinting for alterations in core metabolic processes. To further investigate this hypothesis, the differential expression profiles of proteins associated with respiration, translation and β-oxidation were analyzed (Figure 6).

Significantly, while Figures 4, 5 established that both evolved strains converge on a sessile lifestyle with simplified membranes, Figure 6 reveals a key divergence in how M2C19 and M3C22 support growth on β-myrcene at the level of core metabolism. M2C19 enhances respiration, translation, and β-oxidation capacity relative to wild-type M1, whereas M3C22 systematically downregulates these same processes. This metabolic divergence provides the mechanistic basis for the distinct growth phenotypes and suggests that adaptation to hydrophobic carbon sources can proceed through either metabolic intensification or metabolic conservation strategies, probably also associated with different levels of cell aggregation, as depicted in Figure 4A. The growth phase dynamics reinforce this interpretation. M3C22 shows partial recovery of respiratory and β-oxidation capacity from OD 0.5 to OD 0.8, suggesting that metabolic flux divergence is most pronounced during early adaptation to β-myrcene. M2C19, by contrast, shows increasing expression of core metabolism-associated proteins from OD 0.5 to OD 0.8 (particularly CyoB, Ndh, FadA, FadJ), indicating progressive metabolic intensification as growth proceeds.

## 4 Discussion

### 4.1 Convergent evolution of *fleQ* mutations as a central adaptive strategy

The most striking finding from this ALE approach is the independent acquisition of mutations in the global transcriptional regulator FleQ by most of the retrieved β-myrcene-evolved isolates (7 o out of 8), including M2C19 and M3C22 that were under detailed analysis in this study (Figure 1). To our knowledge, this is the first report of FleQ as a convergent evolutionary target during monoterpene adaptation. This convergent targeting of the same regulator represents compelling evidence for strong positive selection, consistent with theoretical predictions that beneficial mutations in global regulators can provide large fitness gains by simultaneously affecting multiple downstream processes (Tenaillon et al., 2012; Barrick and Lenski, 2013). In M2C19 and M3C22, mutations were localized in the AAA + ATPase core domain, a region essential for ATP-dependent oligomerization and σ^54^-RNA polymerase recruitment (Jyot et al., 2002; Shingler, 2011; Matsuyama et al., 2016). The L211Q substitution in M3C22 affects the Walker A motif responsible for ATP binding, while the +RLHR insertion at position 222 in M2C19 disrupts the Walker B motif required for ATP hydrolysis. These mutations likely compromise the conformational changes necessary for transcriptional activation, effectively decoupling FleQ from its downstream regulon. This interpretation is supported by the fact that, while the FleQ protein itself remains detectable, its target genes, including those of the polar flagellar apparatus and the chemotaxis machinery, are nearly abolished in both evolved strains (Figure 4).

FleQ functions as a c-di-GMP-responsive switch that coordinates the transition between motile and sessile lifestyles in *Pseudomonas* species (Hickman and Harwood, 2008; Baraquet and Harwood, 2013). In the absence of c-di-GMP, FleQ activates flagellar operons while upon c-di-GMP binding, it redirects transcription toward biofilm-associated functions including exopolysaccharide production (Matsuyama et al., 2016). The concurrent downregulation of c-di-GMP phosphodiesterases (DipA, BifA, MorA) in evolved strains suggests potentially elevated intracellular c-di-GMP levels, which would normally reinforce biofilm formation. However, the proteome data suggest that evolved strains do not adopt a classical biofilm state, as evidenced by reduced abundance of exopolysaccharide biosynthesis machinery. Instead, the data support a constitutive “sessile-like” regulatory configuration characterized by stable motility loss without full biofilm commitment: a phenotype that persists even during growth on the non-selective substrate lactate.

Three complementary factors likely explain the strong selection for FleQ inactivation. First, flagellar biosynthesis and operation impose substantial metabolic costs. The flagellar system accounts for approximately 2% of total cellular protein, while motor rotation consumes proton motive force that competes directly with ATP synthesis (Macnab, 2003; Soutourina and Bertin, 2003). By eliminating these energy-intensive systems, evolved strains can redirect resources toward β-myrcene catabolism, propionyl-CoA processing, and stress management. Second, under the homogeneous laboratory conditions of ALE, where β-myrcene is continuously available without spatial gradients, chemotaxis provides no selective advantage. Indeed, non-motile cells may benefit from prolonged residence at oil-water interfaces where β-myrcene partitions, effectively increasing substrate accessibility. As a whole, both evolved strains adopted a permanent lifestyle shift, via FleQ mutations, from motile to aggregated growth, which enhanced β-myrcene catabolic performance. Third, a previous transcriptomic analysis of wild-type *Pseudomonas* sp. M1 during β-myrcene growth revealed that 50 of 56 motility-associated COG genes were downregulated (Soares-Castro and Santos, 2014). Although not explored at the time, this observation suggests that even wild-type M1 transiently suppresses motility and shifts toward a sessile-like state during β-myrcene catabolism. The convergent FleQ mutations identified here may therefore represent a genetic fixation of this transient regulatory response, resolving an inherent conflict between two competing demands: chemotactic motility to locate β-myrcene droplets versus sessile growth to efficiently biotransform the substrate. Thus, evolved strains bypass this dynamic trade-off entirely, committing to a sessile lifestyle by constitutively inactivating FleQ, that, possibly, under laboratory cultivation conditions with agitation, imposes no penalty for substrate access.

### 4.2 Divergent metabolic strategies: Balancing catabolic flux with redox and pH homeostasis

Despite sharing convergent FleQ mutations, M2C19 and M3C22 evolved different metabolic strategies for β-myrcene utilization. Both strains accumulated mutations in other regulatory targets: each evolved strain has mutations in histidine kinase proteins and M3C22 has a mutation in HupB regulator. The unique A57V substitution in the nucleoid-associated protein HU-beta HupB in M3C22 may contribute to mitigating the physiological consequences of organic acid accumulation during β-myrcene catabolism. HU proteins shape chromosome architecture, modulate DNA topology, and thereby influence global gene expression patterns (Remesh et al., 2020; Verma et al., 2023), providing a plausible route by which a single HupB substitution could shift the balance between catabolic flux, energy metabolism, and stress physiology. In *Helicobacter pylori*, HU overexpression protects DNA from acid-induced damage and is required for survival under low pH conditions (Almarza et al., 2014). While the specific biochemical consequences of the A57V substitution remain to be characterized, the observed reduction in respiratory (Figure 6) and translation proteins (e.g., RplA, RplN, RplS, RplK) is consistent with a lower metabolic/energetic throughput that could limit acid-generating flux and thereby attenuate intracellular acidification during β-myrcene catabolism. Together, these observations support the idea that different combinations of accumulated mutations can drive divergent downstream adaptive trajectories under the same selective pressure. Notably, different cell aggregate levels were observed for the evolved strains (Figure 4B), suggesting that the uniquely accumulated mutations may have implications in such trait. Nonetheless, future work (e.g., HupB complementation) will be required to establish whether A57V is causal for the transcriptional and metabolic differences.

M2C19 exhibits rapid and robust induction of the β-myrcene genomic island (GI) and methylcitrate cycle (PRP) from early exponential phase (Figure 2). This high-flux strategy generates substantial quantities of pathway intermediates, with myrcenoic acid accumulating at 10.6-fold higher levels than wild-type M1 (Figure 1B). While this rapid conversion evidences enhanced biotransformation capacity, it creates two interconnected physiological challenges: redox imbalance from accelerated β-oxidation and cytoplasmic acidification from organic acid accumulation. The β-oxidation of β-myrcene-derived acyl-CoA intermediates generates NADH at each dehydrogenation step. Under high catabolic flux, NADH production can exceed the capacity of the electron transport chain to regenerate NAD^+^, creating a potential bottleneck that would stall further catabolism. M2C19 maintains elevated levels of both the high-affinity cbb_3_-type oxidase (CcoNOP) and the high-throughput bo_3_-type oxidase (CyoAB) (Figure 6), suggesting that the electron transport chain operates near capacity during rapid β-myrcene catabolism. In agreement, the induction of nitrate reductase (NarGH; +3.72 log_2_FC for NarG), in M2C19, despite the absence of nitrate in the growth medium, represents an apparent decoupling of gene expression from metabolic function. We hypothesize that this reflects the evolutionary history of regulatory circuits: in natural environments, oxygen limitation typically co-occurs with nitrate availability as an alternative electron acceptor. The regulatory systems that sense low oxygen may therefore be wired to anticipate nitrate presence. In laboratory medium lacking nitrate, oxygen limitation within M2C19 aggregates triggers the expected regulatory response, but the induced enzymes cannot function. This expression-function decoupling illustrates how regulatory programs optimized for natural environments may produce apparently futile responses under artificial conditions. In agreement, a previous transcriptomic analysis of *Pseudomonas* sp. M1 during β-myrcene growth highlighted the induction of what was called anaerobioses-like physiology, based on the upregulation of Dnr-like regulators, the denitrification operon *narK1K2GHJI,* and the Anr-dependent operon *arcDABC* (Soares-Castro and Santos, 2014).

In contrast, M3C22, probably as a consequence of a more extended level of cell aggregation (as depicted in Figure 4A), evidences reduced MyrT, reduced β-oxidation, and diminished respiratory capacity, maintains lower flux and accumulates less acid despite its reduced capacity to oxidize NADH, particularly in the early exponential phase. Thus, M3C22 lifestyle shift essentially contributes to prevent the bottleneck by limiting entry rather than by expanding catabolic capacity during early growth stages, possibly justifying the different levels of accumulated acid intermediates registered (Figure 1D).

### 4.3 Surface remodeling supports specialized monoterpene utilization

The convergent loss of motility in evolved strains is accompanied by broader reconfiguration of the cell envelope (Figure 5). Core membrane functions, including outer membrane integrity (OprF and Pal), protein translocation (SecF and PpiD), and β-barrel assembly (BamBD), remained stable across all strains. This indicates that envelope homeostasis is maintained. However, substantial changes occur in transport systems, surface receptors, and efflux machinery.

Both evolved strains show reduced abundance of the TolC-family efflux protein AggA and the RND-family adaptor AcrA, which typically function in broad-spectrum xenobiotic efflux. This apparent reduction in general efflux capacity may seem counterintuitive given the need to export accumulated organic acids. However, it may reflect a shift from generalist efflux systems toward more specialized transporters better suited to the specific intermediates of β-myrcene catabolism. The strain-specific upregulation of membrane proteins in M2C19, including nutrient transporters (Sbp), lipid homeostasis factors (LolA, VacJ), and metal-handling proteins (CopI), suggests customized envelope adaptations that support its high-flux metabolic strategy. The coordinated reduction of chemotaxis receptors (Aer-2, MCPs) complements the loss of flagellar motility, together eliminating the energetic and biosynthetic costs of environmental sensing and directed movement. In the context of continuous laboratory cultivation with homogeneous substrate availability, these functions provide no selective benefit. The resources freed by eliminating motility and chemotaxis can instead support the specialized catabolic and stress-response systems required for efficient β-myrcene utilization.

### 4.4 Implications and future directions

The parallel evolution of M2C19 and M3C22 provides several insights relevant to both fundamental evolutionary biology and applied biotechnology. From an evolutionary perspective, the convergent targeting of FleQ demonstrates that regulatory mutations can serve as “master switches” that simultaneously affect multiple phenotypic traits. This has implications for understanding how microbial populations adapt to novel environments where initial mutations in global regulators may create permissive conditions for subsequent diversification.

A particularly noteworthy observation is the retention of MyrS, the GI-encoded methyl-accepting chemotaxis protein responsible for β-myrcene sensing, despite the strong reduction of core signal transduction components CheA and CheW in both evolved strains. We hypothesize that this uncoupling reflects adaptation to laboratory cultivation conditions rather than a loss of sensing function. Under orbital agitation, mechanical mixing continuously promotes cells-β-myrcene droplets interactions, bypassing the need for active chemotactic swimming. In this permissive environment, the energetic cost of maintaining flagellar motility and full chemotactic capacity provides no selective advantage, while MyrS retention may still contribute to substrate recognition and efficient attachment at the oil-water interface. Critically, this implies that the phenotypic differences between wild-type and evolved strains observed here may substantially underestimate the true magnitude of adaptation: under static conditions, wild-type M1 would depend on functional chemotaxis to locate dispersed β-myrcene, whereas evolved strains lacking directed motility would be spatially constrained. Experimental validation of this hypothesis would clarify whether the adaptive benefits of motility loss depend on cultivation regime, informing both our mechanistic understanding and the industrial deployment of evolved strains.

Several additional questions remain for investigation. The stability of evolved phenotypes under relaxed selection is unknown: would reversion occur during prolonged cultivation without β-myrcene? Cultivation under static conditions, or in structured habitats mimicking natural environments where substrate is spatially heterogeneous, would reveal the true fitness costs of motility loss and test whether MyrS retention provides a measurable advantage for initial substrate colonization. Additionally, whether similar regulatory mutations targeting FleQ would arise during adaptation to other monoterpenes (limonene, pinene, geraniol) would test the generality of this master regulator as an evolutionary hotspot for terpene tolerance. Complementation studies restoring wild-type FleQ, or deletion of MyrS in evolved backgrounds, would directly test the functional consequences of the mutations identified here.

From a biotechnological perspective, these results identify specific engineering targets for improving microbial monoterpene biotransformation. Targeted inactivation of FleQ could provide immediate gains by redirecting cellular resources toward catabolism without extensive pathway engineering. However, our findings suggest that the benefits of such modifications may be context-dependent: in well-mixed bioreactors, non-motile strains should perform optimally, whereas applications involving biphasic systems with poor mixing or immobilized cells may require retention of chemotactic capacity for efficient substrate access. The divergent strategies of M2C19 and M3C22 further suggest that different applications may benefit from different metabolic configurations: processes requiring rapid conversion might employ M2C19-like modifications (enhanced respiratory capacity, active efflux), while processes sensitive to organic acid accumulation might favor M3C22-like modifications with moderated catabolic flux.

### 4.5 Limitations

Several limitations of this study should be acknowledged. First, while proteomics provides comprehensive coverage of highly abundant proteins, low-abundance regulatory proteins may be underrepresented. Second, the mechanistic basis of specific *fleQ* mutations in the evolved strains requires further characterization. Third, this study focused on only two evolved isolates from the ALE experiment. Analysis of additional independent lineages could strengthen conclusions about convergent evolution. Finally, while correlation between proteomic and metabolomic changes supports our interpretations, additional biochemical validation of key enzymes would provide direct evidence for enhanced catalytic activity.

## 5 Conclusion

Adaptive laboratory evolution of *Pseudomonas* sp. M1 under β-myrcene selection pressure resulted in convergent mutations in the master regulator FleQ, driving a coordinated lifestyle switch from motile to sessile growth. This regulatory rewiring eliminated costly motility systems and enabled divergent downstream metabolic adaptations. Notably, both evolved strains retained the β-myrcene chemoreceptor MyrS despite strong reduction of downstream signal transduction components, suggesting that substrate sensing remains functionally relevant even in the absence of directed motility. We propose that the laboratory cultivation regime (continuous orbital agitation) created a permissive environment for the selection of non-motile variants by mechanically compensating for the loss of chemotactic substrate localization. This implies that the adaptive benefits of FleQ inactivation may be context-dependent, with potentially greater fitness trade-offs in static or spatially heterogeneous environments where chemotaxis is essential. These findings identify FleQ as a key engineering target for monoterpene biotransformation, provide mechanistic insights into how global regulatory mutations drive phenotypic diversification, and highlight the importance of considering cultivation conditions when interpreting laboratory evolution outcomes.

## Data Availability

The mass spectrometry proteomics data have been deposited to the ProteomeXchange Consortium via the PRIDE partner repository with dataset identifier PXD072045. Whole-genome sequencing data are available in NCBI under BioProject PRJNA1405481.
