# Supplementary material for: Multi-omics characterization of β-myrcene-evolved Pseudomonas sp. M1 reveals convergent FleQ mutations and altered catabolic efficiency
Source: Front Mol Biosci. 2026 Apr 13;13:1800048. doi: 10.3389/fmolb.2026.1800048 (PMC13111069; doi:10.3389/fmolb.2026.1800048)

Figure S2. Multidimensional scaling (MDS) ordination of proteome profiles.

Samples are coloured by strain (M1: blue, M2C19: red, M3C22: green), shaped by carbon source (triangle: Myrcene, circle: Lactate), and sized by growth phase (large: OD 0.5, small: OD 0.8). The first two MDS dimensions explain 20.1% and 8.5% of variance, respectively (Supplementary_tables.xlsx, Table_S3).
Samples cluster primarily by carbon source along MDS1, with strain-specific differences visible within each carbon source cluster.

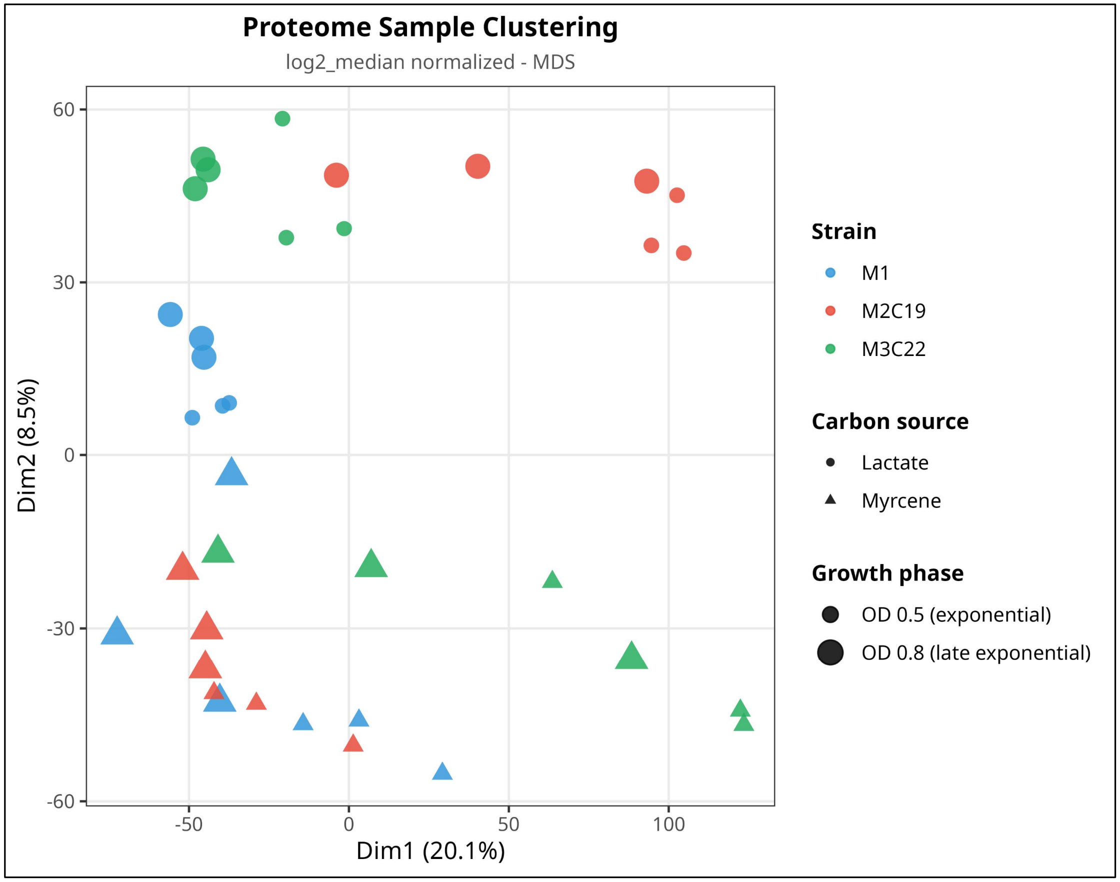

Supplement: Supplementary file 1 [file Image2.pdf]
