# Supplementary material for: Multi-omics characterization of β-myrcene-evolved Pseudomonas sp. M1 reveals convergent FleQ mutations and altered catabolic efficiency
Source: Front Mol Biosci. 2026 Apr 13;13:1800048. doi: 10.3389/fmolb.2026.1800048 (PMC13111069; doi:10.3389/fmolb.2026.1800048)

Figure S3. Protein abundance analysis of the genomic island (GI) and related catabolic clusters.

(A) Boxplots showing normalized $\log_2$ protein intensities for all 21 GI cluster proteins in wild-type M1 and evolved strains (M2C19, M3C22) during growth on β-myrcene (brown) or lactate (pink) at early (OD 0.5) and late (OD 0.8) exponential phase. Individual proteins are overlaid as points. During β-myrcene growth, GI proteins are highly expressed in all strains, with M2C19 showing the highest median abundance. During lactate growth, GI expression is minimal or absent, with evolved strains showing tighter repression than M1.

(B) Heatmap of $\log_2$ spectral counts for individual GI cluster proteins across all conditions. Rows represent proteins (MyrA–T, LuxR regulators); columns represent strain × carbon source × growth phase combinations. Colour scale indicates $\log_2$ spectral count (blue = high, red = low, white = intermediate). Proteins detected only during β-myrcene growth are marked (Myrcene-only). The heatmap illustrates the substrate-specific induction of GI proteins and reveals protein-level differences between strains, particularly the delayed induction pattern in M3C22 at OD 0.5.

(C) Bar plots showing normalized $\log_2$ protein intensities for the Liu cluster (left panels) and PRP operon (right panels) at OD 0.5 (top) and OD 0.8 (bottom). Bars represent mean intensities for each strain × carbon source combination: M1 Myrcene (dark brown), M2C19 Myrcene (medium brown), M3C22 Myrcene (light brown), M1 Lactate (dark pink), M2C19 Lactate (medium pink), M3C22 Lactate (light pink). The Liu cluster shows minimal expression differences between strains. The PRP operon displays β-myrcene-specific induction with M2C19 showing elevated expression at both growth phases, while M3C22 shows delayed induction similar to the GI cluster pattern (Figure 2).

Data for panels A–C are provided in Supplementary_tables.xlsx, Table S4.

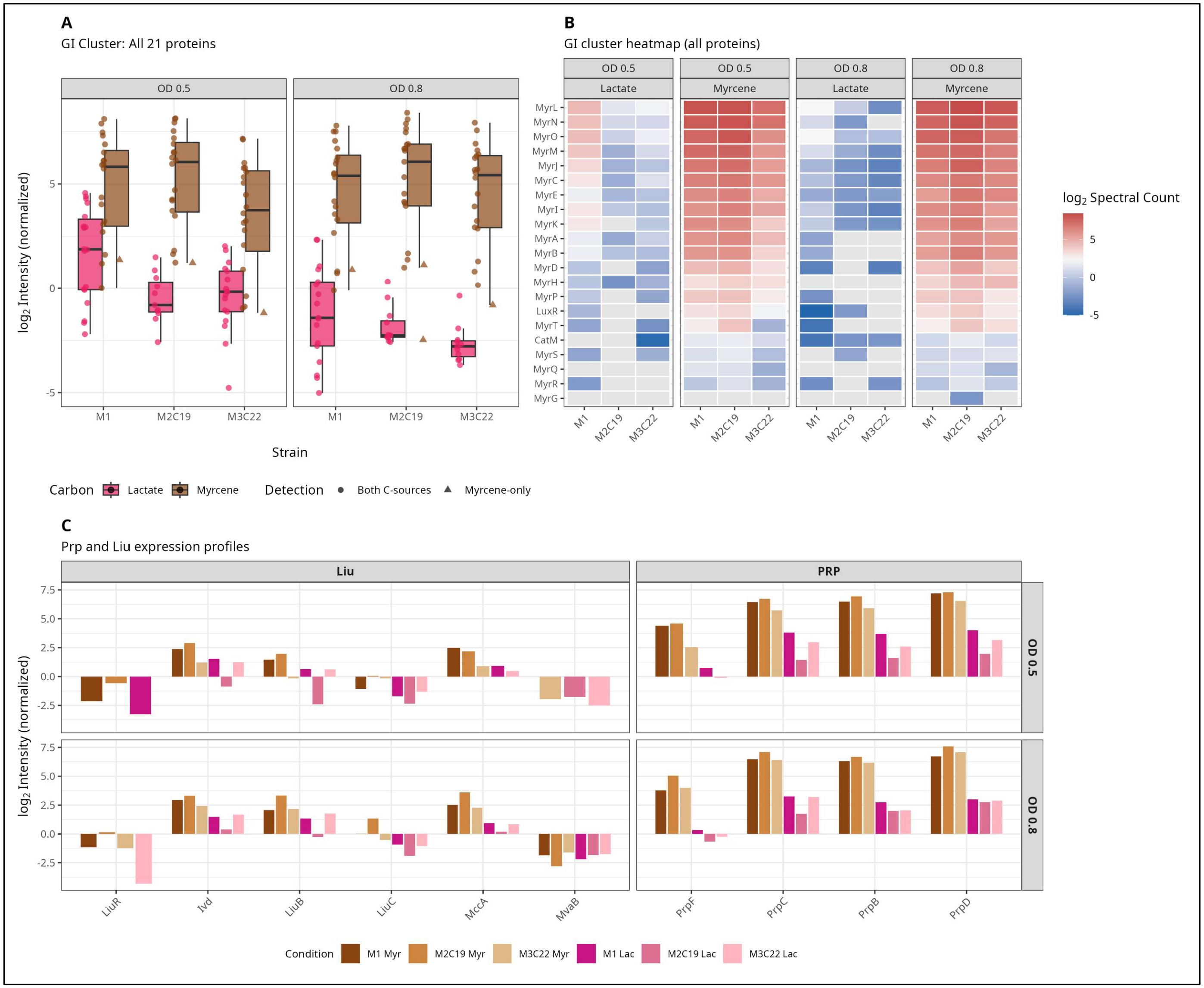

Supplement: Supplementary file 2 [file Image3.pdf]
