# Supplementary material for: Multi-omics characterization of β-myrcene-evolved Pseudomonas sp. M1 reveals convergent FleQ mutations and altered catabolic efficiency
Source: Front Mol Biosci. 2026 Apr 13;13:1800048. doi: 10.3389/fmolb.2026.1800048 (PMC13111069; doi:10.3389/fmolb.2026.1800048)

Figure S1. Schematic overview of the β-myrcene catabolic pathway in *Pseudomonas sp.* M1.

The pathway comprises the genomic island (GI) main pathway and the 2-methylcitrate cycle (PRP). The GI main pathway initiates with the hydroxylation of β-myrcene to myrcen-8-ol by the myrcene hydroxylase MyrHG, followed by sequential oxidation to myrcenal (MyrB, zinc-dependent alcohol dehydrogenase) and myrcenoic acid (MyrA, aldehyde dehydrogenase). Activation to myrcenyl-CoA (MyrC, acyl-CoA synthetase) enables entry into β-oxidation. The first β-oxidation cycle, catalyzed by MyrI/MyrJ (acyl-CoA dehydrogenase), MyrN/MyrD (enoyl-CoA hydratase), MyrN (3-hydroxyacyl-CoA dehydrogenase), and MyrK (thiolase), yields one propionyl-CoA and the C7 intermediate. A second β-oxidation cycle (MyrQ, MyrO, MyrC, MyrL) generates an additional propionyl-CoA and two acetyl-CoA. Propionyl-CoA enters the 2-methylcitrate cycle where it is condensed with oxaloacetate by PrpC (2-methylcitrate synthase) to form 2-methylcitrate, which is subsequently converted to 2-methylisocitrate (PrpF, 2-methylaconitate isomerase) and cleaved to pyruvate and succinate (PrpB, methylisocitrate lyase). Both products enter the TCA cycle.

Overall stoichiometry: one molecule of β-myrcene ($C_{10}H_{16}$) yields two propionyl-CoA and two acetyl-CoA. Box inset shows predicted flux values from FBA/FVA validation (GI: 5.0; PRP: 10.0 mmol/gDW/h). Colour coding: light blue, metabolites; green, GI enzymes; orange, PRP cycle enzymes; dark green, TCA cycle entry points.

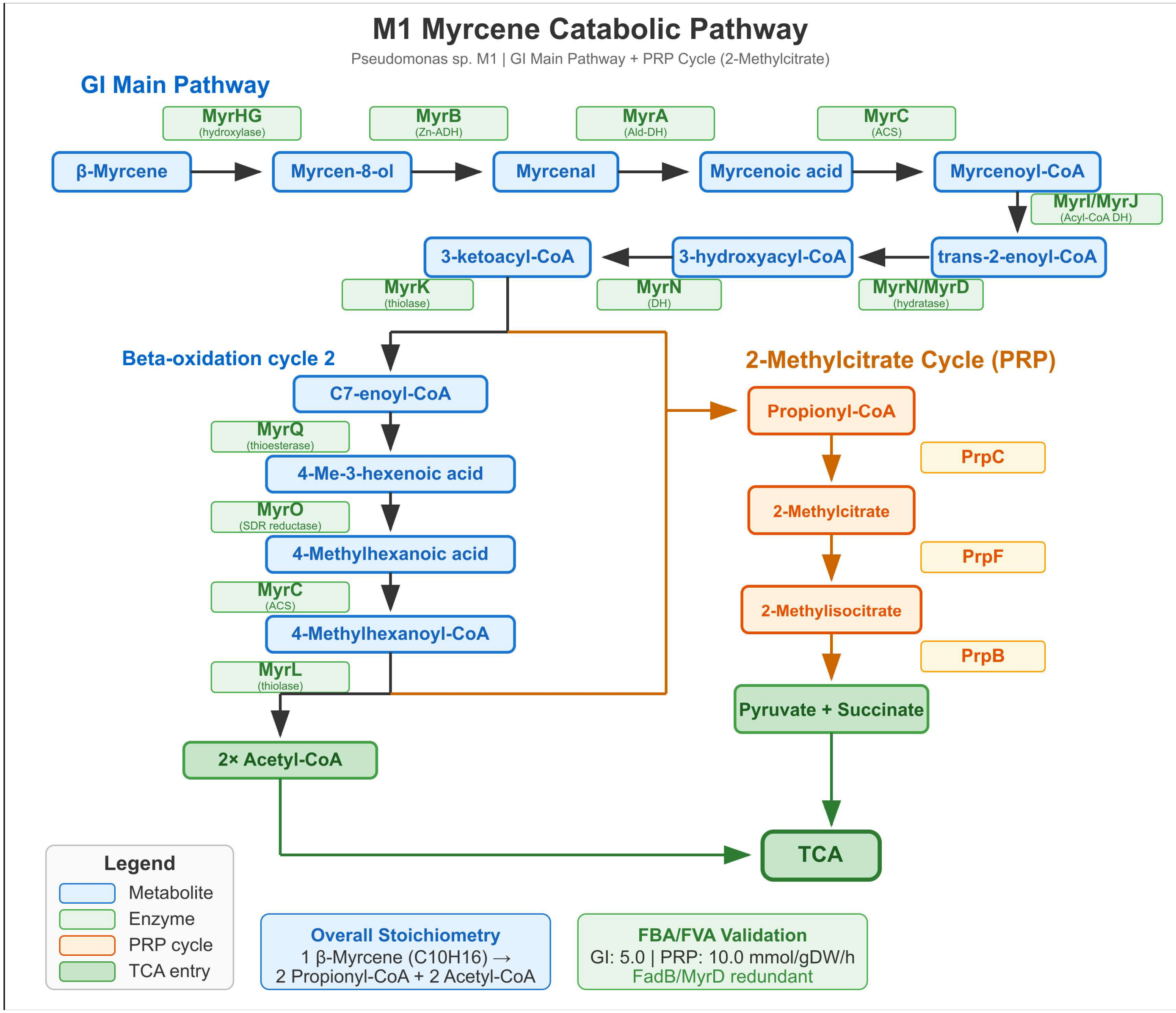

Supplement: Supplementary file 3 [file Image1.pdf]
